# With the Burgher Commandoes in South Africa

**Published:** 1915-03-13

**Authors:** 


### WITH THE BURGHER COMMANDOES IN SOUTH AFRICA.

### My Medical Experiences in the Campaign.

By A COMMANDO DOCTOR.

Not the least of the difficulties that confronted the Government of the Union of South Africa at the outbreak of war was the want of adequate organisation of the medical department of its recently constituted Defence Force. This force is a child of recent birth, scarcely two years old, and its medical branch six months ago was still embryonic.

Fortunately, however, in Lieut.-Colonel Stock the Director of Medical Services, the Union Government possesses a very able and hardworking official, and it is mainly due to his tireless energy that the Union forces can now boast of a medical department that is certainly efficient enough for the work it has to do. Those acquainted with the methods and the resources of the Royal Army Medical Corps can have little idea of the organising difficulties with which Colonel Stock's department has had to contend. To begin with, there was no trained *personnel*; officers as well as men had to be initiated in the work; equipment had to be raked together; stores provided—not the least of the difficulties was the shortage of drugs, some of which, highly necessary for work in this country, such as emetine, were almost unprocurable—transport had to be requisitioned, and the whole area of the Union had to be mapped out into medical districts and properly organised.

### The Burghers' View of Medical Field Service.

A feature of the campaign in this country is the employment of burgher commandoes. Under the old law, in time of national difficulty, the State had the right to call up every able-bodied citizen or burgher for service in the field. The field cornet issued a commandeering note calling upon the burgher to appear, with horse, saddle, bridle, rifle, ammunition, and provisions for three days, at some central point for mobilisation purposes.

### Mobility and Sanitation.

The commandoes so formed are exceedingly mobile; they have little transport, move very rapidly, and are, for work in this country, exceedingly effective. But to provide such mobile forces with adequate ambulances is a matter of great difficulty. During the late war the Boer commandoes in the field had practically no ambulance arrangements; one or more ambulances were attached to separate columns, but when the war became a matter of mounted commando evolutions, the doctor was forced to content himself with a led horse and a pack-saddle. One of the results of this war experienced fourteen years ago is that the average burgher is to-day very contemptuous of medical service in the field. He does not realise that his commando will be co-operating with large forces, and that questions of sanitation, which do not gravely concern a unit that perpetually shifts camp so that it is never on the same spot for longer than a few hours, loom very largely before the medical department. Nor does he realise the importance of hospitals, of ambulances, of first-aid dressings, and of preventive medicine generally as understood in modern armies. All this has to be taught him, and for that reason the doctor who is attached to a commando has a lively and highly interesting and exciting time.

### The Rebels' Ambulance.

So far commando experience has been limited to the rebellion in the three provinces during the latter part of last year. With the rebels there was only one ambulance, a voluntary arrangement undertaken by two medical men on their own responsibility, though with the consent of the Government, and little has so far been made public concerning their activities. With the commandoes, the general arrangement has been to provide each brigade with two doctors and ambulance equipment, consisting of a field ambulance, a buck-waggon, capable of carrying about 5,000 pounds weight, a water-cart, and a few riding-horses. These mobile ambulance units have on the whole worked excellently. In a few instances, however, it was found that they were by no means mobile enough.

At one fight, to give an example, where the percentage of killed and wounded was fairly high, the ambulance stuck in the mud eight miles away from the scene of operations, and the doctors, who had ridden with their commando and were actually in the firing line, were forced to attend the casualties with nothing else but their haversacks and first-aid dressings. A glorious stand-by on such occasions has been the solution of morphia and atropine, which most of us carried ready for immediate use.

An injection of this given to a wounded man on the spot largely obviates shock and renders him more comfortable until he can be moved. Necessarily a long time must elapse, in some cases, before that removal can take place. There is no trained bearer company, and the doctor has to teach the volunteer helpers how to lift the casualty, how to transport him on the improvised stretcher, made out of a couple of tunics and a couple of rifles.

### South African Motor Ambulances.

Very often, moreover, the difficulties of treating the wounded are accentuated by the lack of accommodation and water. In the operations round about Upington, where the district is arid and sterile, the water obtainable only brackish, and the shelter conspicuous by its absence, much difficulty was experienced in evacuating the wounded to the nearest base hospital, as the heavy sand made progression by the ambulances very slow work. Motor ambulances have in many cases done excellent service, but in the summer months, which are here the rainy season, the heavy, sodden turf makes a very poor road for motor-cars, and only the lighter types of car can be employed.

DOCTORS FIRED ON.

During the rebellion the commando doctors were exposed to serious dangers and inconveniences, owing to the peculiar nature of the campaign. Some of them had narrow escapes while in the firing line; since, owing to the difficulty of differentiating them from the burghers—the most charitable reason to suggest—the rebels made no attempt to discriminate between them and combatants. Actual casualties in their ranks there were fortunately none. Yet the experience has been interesting and wholly instructive, though not so varied, perhaps, as fell to the lot of medical men in the last war.

NATURE OF THE WOUNDS.

There are, for instance, no shell wounds, since only on one occasion were the guns used. One or two bayonet wounds have been recorded, but the majority of the wounds have been made by bullets of the Mauser, or Martini, or Lee-Enfield rifle. Many of the rebels used the soft-nosed, old-fashioned Martini sporting bullet, which makes a fairly big exit wound and which is constantly being mistaken for a dum-dum. One of the interesting wounds was made by a round leaden bullet, fired from an elephant gun, an antiquated weapon that made a sound like a small cannon and a wound almost like that inflicted by a fragment of shell. This had shattered a man's humerus in the upper fifth, torn away the entire attachment of the deltoid, pectoralis major and minor, and converted the outer wall of the axilla into a mass of half-minced flesh.

